# Tight and early HbA_1c_ control in patients with type 2 diabetes mellitus in Spain: quantifying the social value

**DOI:** 10.3389/fpubh.2025.1511108

**Published:** 2025-07-11

**Authors:** María Merino, Paulina Maravilla-Herrera, Sara Artola, Javier Escalada, Antonio Pérez, Juantxo Remón, José L. Trillo-Mata, Joan A. Vallès-Callol, Álvaro Hidalgo-Vega

**Affiliations:** ^1^Department of Health Outcomes Research, Weber, Madrid, Spain; ^2^José Marvá Health Centre, Madrid, Spain; ^3^Red de Grupos de Estudio en Atención Primaria de Salud (redGDPS) Foundation, Madrid, Spain; ^4^Department of Endocrinology and Nutrition, Clínica Universidad de Navarra, Pamplona, Spain; ^5^Centro de Investigación Biomédica en Red de la Fisiopatología de la Obesidad y Nutrición (CIBEROBN), Madrid, Spain; ^6^Department of Endocrinology and Nutrition, Hospital de la Santa Creu i Sant Pau, Barcelona, Spain; ^7^Centro de Investigación Biomédica en Red de Diabetes y Enfermedades Metabólicas Asociadas – CIBERDEM, Instituto de Salud Carlos III, Madrid, Spain; ^8^Department of Medicine, Universitat Autónoma de Barcelona (UAB), Barcelona, Spain; ^9^Federación Española de Diabetes, Madrid, Spain; ^10^Pharmacy Service of Health Area Malvarrosa Clinical Department, Conselleria de Sanitat, Valencia, Spain; ^11^Institut Català de la Salut, Barcelona, Spain; ^12^Department of Economic Analysis and Finances, University of Castilla-La Mancha, Toledo, Spain; ^13^Fundación Weber, Madrid, Spain

**Keywords:** socioeconomic impact, type 2 diabetes, glycemic control, monitoring, complications, hospitalizations, quality of life, mortality

## Abstract

**Introduction:**

The aim of this study was to estimate the social value of a tight and early control of patients with type 2 diabetes during the 5 years after diagnosis in Spain, compared to higher hemoglobin A1c (HbA_1c_) goals.

**Methods:**

An economic model based on the scientific literature was used to estimate the 5-year social value of maintaining tight and early type 2 diabetes control, i.e., keeping HbA_1c_ levels <6.5%, during the 5 years after diagnosis in Spain, compared to non-tight control. Areas of analysis included healthcare resource utilization, the presence of complications, quality of life, and mortality. The outcomes corresponding to these two types of control (tight vs. non-tight) were multiplied by their unit cost or financial proxy to obtain the economic impact associated with each type of control. Social value was estimated as the reduction in the economic impact of a non-tight control when tight control is implemented and maintained. The results are expressed in euros for the year 2021.

**Results:**

The economic impact of tight control during the first 5 years after type 2 diabetes diagnosis was estimated to be €1,010 million in Spain (€13,473 per patient), which is lower than the impact of non-tight control, which was estimated to be €1,127 million (€16,122 per patient) during the same period.

**Conclusion:**

Maintaining tight and early control of type 2 diabetes during the first 5 years after diagnosis could generate a positive social value of €2,649 per patient over that period, in terms of better health outcomes, increased quality of life, and decreased premature deaths.

## Introduction

Diabetes Mellitus is a heterogeneous metabolic disorder characterized by chronic hyperglycemia. Among the different types of diabetes, type 2 diabetes is the most prevalent, accounting for more than 90% of all cases worldwide (1).

In Spain, the known incidence of type 2 diabetes is 3.7 cases per 1,000 adults, although the actual incidence could be up to 11.6 cases (2). On the other hand, according to the International Diabetes Federation, the age-adjusted prevalence was estimated at 10.3% in adults (20 to 79 years) in 2021 (1).

Type 2 diabetes is characterized by insufficient insulin secretion and insulin resistance. Different risk factors can contribute to the development of this disease, including age, male sex, non-Caucasian ethnicity, genetic susceptibility, obesity, inadequate diet, physical inactivity, and hypertension or dyslipidemia (1, 3).

This disease is associated with long-term issues due to microvascular (retinopathy, nephropathy, and neuropathy) and macrovascular complications, such as coronary heart disease, cerebrovascular disease, and peripheral vascular disease (1). Accordingly, type 2 diabetes mellitus is the main cause of cardiovascular disease, blindness, limb amputation, kidney failure, and death, and it has a significant impact on the national healthcare system (NHS), patients’ quality of life, and society as a whole (1).

Type 2 diabetes is also associated with a higher risk of mortality (1). In 2019, a total of 9,644 deaths occurred in Spain due to all types of diabetes mellitus, making it the eighth leading cause of death in the country (4). In 2020, this figure increased to 11,297 deaths, although this could be attributed to the COVID-19 pandemic (5). These deaths are primarily caused by the abovementioned complications related to diabetes, with cardiovascular disease being the most prevalent (6).

There is significant room for improvement in the management of type 2 diabetes. Currently, the hemoglobin A1c (HbA_1c_) goal for the majority of patients is set at <7%, while some patients may have a less stringent target (<8–8.5%) (7). However, tight (HbA_1c_ < 6.5%) and early control (within the first 5 years after diagnosis) of type 2 diabetes is one of the strategies associated with the greatest benefits, such as decreased healthcare resource consumption, increased quality of life, and reduced mortality rates (8–10), which translates into social value gain (11), aligning with value-based healthcare models that have a comprehensive view of the socioeconomic impact and contemplate how the patients and their families, the healthcare system, and society at large value interventions (12).

Previous economic evaluations have shown that interventions aimed at achieving tight glycemic control are cost-effective when assessed over long-term time horizons (13). In Spain, improved HbA_1c_ was found to be the main driver of improved clinical outcomes, resulting in an incremental cost-effectiveness ratio well below the willingness-to-pay thresholds of €11,000, €21,000, and €30,000 (14, 15). Therefore, in line with current recommendations (7), maintaining tight and early control of type 2 diabetes may reduce the burden on the NHS, especially in the context of an increasing prevalence of type 2 diabetes.

Estimating the social value gained from tight and early control of type 2 diabetes can provide a more comprehensive understanding of the disease. Evidence based on the social value of interventions considers their impact on patients’ quality of life and society at large, in addition to the most commonly reported impacts, such as improved clinical outcomes and reduced healthcare resource consumption. Therefore, the objective of the present study was to estimate the social value of tight and early control of patients with type 2 diabetes during the 5 years after diagnosis in Spain, compared to higher HbA_1c_ goals.

## Materials and methods

**Figure 1:**
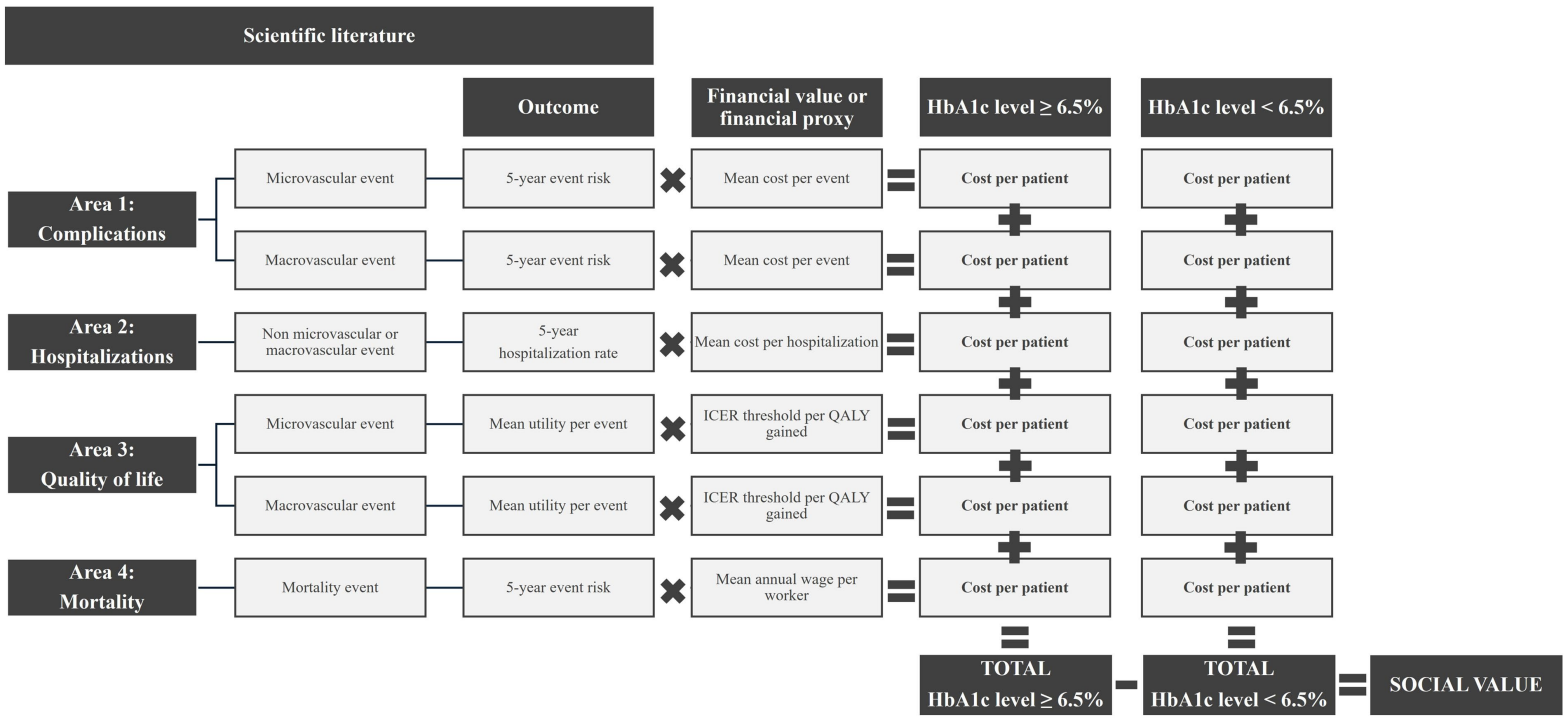
Outline of the economic model.

An economic model with a 5-year time horizon and social perspective was designed and developed using Microsoft Excel to estimate the economic impact of maintaining tight control on type 2 diabetes (HbA_1c_ < 6.5%) during the 5 years after diagnosis and its associated social value, compared to higher HbA_1c_ goals (Figure 1). Following the principles of social value adopted in the present study (16), a 5-year time horizon was selected as the most conservative option to capture the minimum social value that would be generated by early and tight control of type 2 diabetes, as the association between the duration and intensity of early glycemic exposure and the risk of complications did not change when follow-up was right censored at 5 years of diagnosis (8). Moreover, longer disease durations could make it difficult to control diabetes (7).

The economic model was developed using a mixed-methods approach. Three online meetings were conducted with an advisory committee of six experts, representing the main stakeholders in type 2 diabetes care in Spain (including primary care, endocrinology, hospital pharmacy, healthcare management, and patients). These meetings aimed to develop the search strategy for the literature review, to discuss and agree on the most appropriate data to include in the economic model, and to validate the results of the analysis.

To identify the clinical and social impacts related to glycemic levels, a non-systematic scientific literature review was carried out on the PubMed® search engine, combining search terms related to the disease (type 2 diabetes mellitus, HbA_1c_, and tight control) and its impact (burden, costs, expenditures, health care cost, direct cost, indirect costs, resource utilization, health care utilization, informal care, productivity, economic, QALY, visual aids, technical aid, and social value), including relevant synonyms. This search retrieved 134 articles, of which only 10 provided relevant data necessary to achieve the objective of the present study.

Based on the results of the literature review, four areas were considered in the analysis, as these were the only ones with valid information for this study: (1) the impact on the consumption of healthcare resources related to micro- and macro-vascular complications, (2) hospitalizations due to type 2 diabetes (excluding those linked to micro- and macro-vascular complications), (3) loss of quality of life due to micro- and macro-vascular complications, measured in quality-adjusted life years (QALYs), and (4) the impact on mortality, monetized as labor productivity losses due to premature deaths.

The economic impact was estimated by multiplying the outcomes associated with each type of HbA_1c_ control by their corresponding financial values or proxies (unit costs) within each area of analysis (Table 1 and Supplementary Figure 1). To estimate the costs of non-tight control (HbA_1c_ ≥ 6.5%), available data on different HbA_1c_ levels were used and weighted by the corresponding incidence of type 2 diabetes within each level (Table 2).

**Table 1:** Outcomes according to area of analysis, HbA_1c_ level, and the corresponding financial values or proxies.

Areas of analysis and outcomes	<6.5%	6.5 to <7%	7 to <8%	8 to <9%	≥9%	Financial values or proxies	Unit cost (€)
Complications							
Probability of having a microvascular event in the first five years since diagnosis (8)	0.043	0.055	0.062	0.091	0.177	Average cost per hospitalization due to a microvascular event^d^ (48)	4,294.30
Probability of having a macrovascular event in the first 5 years since diagnosis (8)	0.226	0.249	0.285	0.287	0.361	Average cost per hospitalization due to a macrovascular event^e^ (48)	3,748.06
Hospitalizations							
Annual hospitalization probability not linked to micro- or macro-vascular events^a^ (9)	0.162 (<7%)		0.240 (≥7%)			Average cost per hospitalization due to type 2 diabetes (48)	2,405.72
Loss of quality of life							
Average utility linked to microvascular events^b^ (8, 49)	0.846	0.843	0.841	0.834	0.814	Incremental cost-effectiveness threshold per QALY gained (15)	21,000.00
Average utility linked to macrovascular event ^c^ (8, 49)	0.820	0.816	0.810	0.810	0.798		
Mortality							
Probability of death in the first 5 years since diagnosis (8)	0.058	0.062	0.072	0.091	0.102	Average annual earnings per worker in Spain (50)	22,837.59

^a^Data available only for patients with HbA_1c_ < 7% and HbA_1c_ ≥ 7%. ^b^Data calculated from the average utility associated with microvascular events (late-stage kidney disease (51), amputation (51), and diabetic retinopathy (52)) weighted by the proportion of patients having at least one microvascular event (8) and the average utility in people with diabetes mellitus without macrovascular events (used as a proxy for the average utility in patients without microvascular events) (49) weighted by the proportion of patients without microvascular events (8). ^c^Data calculated from the average utility in people with diabetes mellitus with macrovascular events (high blood pressure, other heart diseases, myocardial infarction, stroke, cerebral infarction, cerebral hemorrhage) weighted by the proportion of patients having at least one macrovascular event (8), and the average utility in people with diabetes mellitus without macrovascular events (49) weighted by the proportion of patients without macrovascular events (8). ^d^Average cost of the following causes: E11.22-type 2 diabetes with chronic diabetic nephropathy, E11.3-type 2 diabetes with ophthalmic disease (includes all subsections), and 305 amputation of lower extremities except the toes. ^e^Average cost of the following causes: intracranial hemorrhage, stroke and precerebral occlusions with infarction, non-specific stroke and precerebral occlusions without infarction, transient ischemic attack, acute myocardial infarction, acute and subacute endocarditis, heart failure, cardiac arrest and shock, peripheral vascular disorders and others, coronary arteriosclerosis and angina pectoris, hypertension, cardiac arrhythmias and conduction disorders, and cardiomyopathy. HbA_1c_, hemoglobin A1c; QALY, quality-adjusted life years.

**Table 2:** New cases of type 2 diabetes per year according to their HbA_1c_ levels.

	<6.5%	6.5 to <7%	7 to <8%	8 to <9%	≥9%	Total
Percentage weight with respect to the total number of patients according to HbA_1c_ levels (8)	51.8%	21.3%	17.1%	5.1%	4.7%	100%
Percentage weight with respect to the total number of patients according to non-tight HbA_1c_ levels (≥6.5%)	N/A	44.1%	35.5%	10.6%	9.7%	100%
New annual cases of type 2 diabetes in Spain	74,985	30,847	24,827	7,443	6,771	144,873 (2,17)

The percentage weight of patients with non-tight control (≥6.5%) is weighted by their relative incidence. HbA_1c_, hemoglobin A1c; N/A, not applicable.

Subsequently, the social value was estimated by subtracting the economic impact of maintaining tight control from that of non-tight control. Both the economic impact and the social value were calculated for a period of 5 years and reported per patient and at a population level, taking into account the estimated new annual cases of type 2 diabetes in Spain (144.873) (2, 17). While the social value per patient is defined as the difference in the economic impact between maintaining tight control and non-tight control, the potential social value at a population level was estimated based on the reduction in the economic impact generated by all patients with non-tight control if they had maintained tight control. The results are expressed in euros in 2021. Unit prices before 2021 were updated according to the corresponding general or medical consumer price index (18).

A sensitivity analysis was carried out to assess the strength of the model by varying specific data points (Supplementary Table 1). Therefore, three scenarios are presented: the base (reference) scenario, the lower limit or best scenario (the one that would result in a lower economic impact), and the upper limit or worst scenario (the one that would result in a greater economic impact). The different scenarios were configured based on the confidence intervals of the data used or on different assumptions previously validated by the expert committee.

Given the nature of this study, approval by an institutional review board or ethical review board was not required. Nevertheless, the study procedures were conducted in accordance with the Declaration of Helsinki (1975/83).

## Results

**Figure 2:**
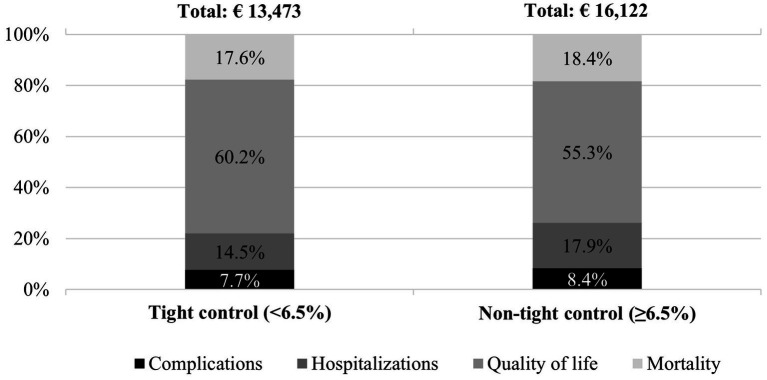
Economic impact and its distribution by area of analysis and HbA_1c_ control per patient. HbA_1c_, hemoglobin A1c.

The economic impact of tight control of type 2 diabetes during the first 5 years after diagnosis was estimated to be € 1,010 million in Spain (€ 13,473 per patient). This impact was lower than that of a non-tight control, which was estimated at € 1,127 million (€ 16,122 per patient) during the same period (Table 3), despite the larger number of type 2 diabetes tight-control cases (74,985) compared with non-tight control cases (69,888) among the new annual type 2 diabetes diagnoses (Table 2). According to Figure 2, quality of life accounts for most of the economic impact, representing more than half of the total impact, followed by mortality, hospitalizations, and complications. The latter represents less than 10% of the total cost.

**Table 3:** Economic impact and social value by area of analysis and HbA_1c_ control, per patient and in Spain.

Area of analysis	Economic impact/social value	HbA_1c_ control	Base scenario	
			Per patient (€)	Spain (€)
Complications	Economic impact	Tight control^a^	1,034.5	77,568,395
		Non-tight control	1,352.5	94,526,210
	**Social value**		**318.1**	**22,230,033**
Hospitalization	Economic impact	Tight control	1,949.6	146,189,110
		Non-tight control	2,890.0	201,974,790
	**Social value**		**940.4**	**65,721,955**
Loss of quality of life	Economic impact	Tight control	8,114.2	608,439,011
		Non-tight control	8,918.7	623,310,065
	**Social value**		**804.5**	**56,225,827**
Mortality	Economic impact	Tight control	2,374.9	178,082,783
		Non-tight control	2,961.1	206,944,660
	**Social value**		**586.2**	**40,965,921**
Total	Economic impact	Tight control	13,473.1	1,010,279,299
		Non-tight control	16,122.3	1,126,755,724
	**Social value**		**2,649.1**	**185,143,736**

The social value at a population level corresponds to the total reduction in the economic impact that could be obtained if all patients with non-tight control in Spain had tight control. ^a^The data do not vary between the sensitivity analysis scenarios. HbA_1c_, hemoglobin A1c.

According to the sensitivity analysis, the economic impact of maintaining tight control of type 2 diabetes over the first 5 years since its diagnosis remains lower than that of non-tight control in both alternative scenarios. In the lower limit, the total economic impact of maintaining tight control was estimated at € 404 million (€ 5,388 per patient), lower than that of a non-tight control, estimated at € 468 million (€ 6,696 per patient) in Spain. For the upper limit, maintaining a tight control was estimated to have an impact of € 2,337 million (€ 31,170 per patient), while the impact of a non-tight control was estimated at € 2,657 million (€ 38,021 per patient) in Spain (Supplementary Table 2 and Figure 2).

In terms of social value, maintaining tight control of type 2 diabetes over the first 5 years from diagnosis would generate a positive social value of € 2,649 per patient over that period (Table 3). Considering the different areas of analysis, reducing hospitalizations would account for 35.5% (€940) of the total social value generated. Therefore, improving quality of life would account for 30.4% (€ 805) of the total social value generated. Moreover, work productivity gains due to reduced mortality would account for 22.1% (€ 586) of the total social value generated. Finally, reducing the complications of type 2 diabetes would account for 12.0% (€ 318) of the total social value generated.

If non-tightly controlled type 2 diabetes patients in Spain had tight control over the first 5 years after diagnosis, a social value of € 185 million could be generated, which represents an 8.7% reduction in the total economic impact of type 2 diabetes in Spain (Table 3).

In Spain, reducing hospitalizations would generate the highest social value (€ 65.7 million), which represents a reduction of 18.9% over its total cost. Thereafter, improving quality of life would generate a social value of € 56 million, which represents a reduction of 4.7%, being an especially low proportion given that the largest impact of type 2 diabetes was on quality of life. Moreover, the social value related to reduced mortality, thereby avoiding work productivity losses, would amount to € 41 million, which represents a reduction of 10.6% over its total cost. Finally, the social value of reducing micro- and macrovascular complications would amount to € 22.2 million, which represents a reduction of 12.9% over the total cost.

According to the sensitivity analysis, the potential social value of maintaining a tight control compared with a non-tight control could range between € 1,309 and € 6,852 per patient over a period of 5 years since diagnosis, which translates into a range of € 92 million to € 479 million in Spain (Supplementary Table 2).

## Discussion

Maintaining a tight and early control of type 2 diabetes during the first 5 years after diagnosis could have a lower economic impact and, therefore, greater social value, not only in terms of health outcomes but also in terms of increased quality of life and decreased premature deaths. According to the results of this study, the potential social value that could be generated by a tight and early control of type 2 diabetes would amount to € 185 million and represent 8.7% of the total economic impact of this disease in Spain. In the worst-case scenario, a social value of € 91.5 million will be generated. The analysis of the social value related to type 2 diabetes management allows for a more comprehensive view of the impact of the disease on patients, the NHS, and society at large.

Considering areas of analysis, hospitalizations for type 2 diabetes (not linked to micro- or macrovascular events) would generate the highest social value in the first 5 years after diagnosis (€ 65.7 million), representing 18.9% of the total cost in Spain. Various studies have shown a significant association between HbA_1c_ levels and the number of hospitalizations in people with type 2 diabetes, revealing that maintaining a tight and early control would result in fewer hospitalizations compared with a non-tight control (9, 19).

The complications associated with type 2 diabetes are probably among the most important factors associated with disease progression (20). Various studies have indicated that glycemic levels are directly related to the development of micro- and macrovascular complications (21, 22), and that maintaining tight and normoglycemic levels could reduce the risk of these complications (8). Accordingly, the results of the present study showed that maintaining a tight and early control of glycemic levels could generate a social value of € 22.2 million in Spain, representing 12.9% of the total cost of this area.

The cost of mortality focuses on the associated loss of work productivity. According to the literature, maintaining a tight and early control of type 2 diabetes can reduce the risk of mortality (8, 23). According to our results, the social value related to the reduction in mortality would be € 41.0 million in Spain, representing a reduction of 10.6% over the total cost for this area of analysis.

Quality of life is the area of analysis with the highest associated cost, but its social value represents only 4.6% of the total cost in Spain (€ 56.2 million). While the present study considers the impact of micro- and/or macrovascular events on the loss of quality of life, some studies suggest that poor control of type 2 diabetes may also be related to the presence of depression, which would have a significant impact on patients’ quality of life (10, 24, 25). Moreover, the patients’ perception of achieving normoglycemia is positive, as they stated that it would mean an improvement in the physical and psychological aspects of their lives (26). Therefore, the potential social value of this area may be underestimated.

Nevertheless, tight and early control of type 2 diabetes poses different challenges in the Spanish context. First of all, there is the trivialization of the disease due to its chronic condition. Over time, patients’ concerns about the impact of the disease on their health diminish. Type 2 diabetes is further trivialized when compared with a diagnosis of type 1 diabetes, as the latter is considered more serious than the former in both social and healthcare settings for several reasons, one being that the diagnosis of type 2 diabetes is generally imprecise and superficial (e.g., communicating the diagnosis by saying “you have sugar in your blood”), downplaying the severity of the disease (27). This may determine how the patient perceives the diagnosis and disease throughout their lifetime. Accordingly, as opposed to patients with type 1 diabetes, type 2 diabetes has been shown to be significantly associated with worse perceptions of illness, self-management, and self-monitoring of blood glucose, which determine the risk of complications and health outcomes (28). Therefore, primary diabetes prevention strategies should focus on risk communication at the population level as well as in primary care practice to be effective (29, 30), as individuals’ perception of type 2 diabetes is modifiable and may improve glycemic control (31). Moreover, self-management tools offered by the healthcare system to patients with type 2 diabetes (mainly to non-insulinized patients) are much scarcer than those offered to patients diagnosed with type 1 diabetes at the time of diagnosis and during follow-up (32). Therefore, empowering patients with type 2 diabetes to not only understand the disease and its short- and long-term impact but also enhance treatment adherence and healthy lifestyles is extremely important. In this context, the pharmacological approach is turning less glucocentric to focus on additional functions such as weight control and cardiorenal protective effects (8, 33). Additionally, type 2 diabetes management is affected by a certain degree of therapeutic inertia (34), as compared with stronger treatment decisions made for type 1 diabetes. In Spain, the prevalence of clinical inertia regarding type 2 diabetes ranges from 18.1 to 60%, hampering appropriate management of glycemia (35). This may delay treatment modifications between 1 and 5 years in patients with type 2 diabetes achieving the target HbA_1c_ level with diet and exercise alone (35).

According to the American Diabetes Association (ADA) (7), the approach to diabetes management should be aligned with the Chronic Care Model (CCM), which emphasizes person-centered care, integrated long-term treatment approaches to diabetes and comorbidities, and ongoing collaborative communication and goal setting between all team members. Primary care physicians and nurses play a central role in effective management and care integration. However, other members, such as diabetes specialists, diabetes educators, dietitians, podiatrists, and pharmacists, are important to effectively care for patients with diabetes. Moreover, institutional resources are necessary for the prevention of type 2 diabetes in the general population, and patient associations may play an important role in patient education regarding attitude and behavioral changes (32, 36).

This study has some limitations. The results should be interpreted with caution. First, although previous studies have addressed the potential impact of type 2 diabetes on health outcomes in the Spanish population (27, 37, 38) and the impact of risk factors on its incidence (39), there is a lack of scientific evidence related to the tight control (HbA_1c_ < 6.5%) of type 2 diabetes in Spain. Accordingly, available data were assumed to be representative of the Spanish population, introducing potential bias into the results. Despite this limitation, the studies selected for this analysis were considered homogeneous and equivalent within each area analyzed. Moreover, this bias, together with publication bias, was minimized by using the most conservative data available in all cases (i.e., those that would result in the smallest possible saving), validating assumptions with a multidisciplinary advisory committee of 6 experts based on their knowledge and experience, and performing a sensitivity analysis to test the strength of the results upon data uncertainty. Second, data on the impact of tight type 2 diabetes control on hospitalizations were not available according to the HbA_1c_ < 6.5% definition. Alternatively, an HbA_1c_ level of <7% was assumed as the tight control in this area. Third, data related to other kinds of impacts, such as acute complications, psychological distress, or erectile dysfunction in men, were lacking and hence not considered (40, 41). Nevertheless, the results of the present study may be used to guide future, more comprehensive studies. Fourth, assumptions related to the moment at which an event or death occurs were made, which may have introduced some uncertainty into the results. In the reference scenario, the event or death would occur 2.5 years after the diagnosis, halfway through the time horizon, affecting the following 2.5 years of the time horizon; in the lower bound (best scenario), the event or death would occur at the beginning of year 5, affecting only the last year of the time horizon; and in the upper bound (worst scenario), the event or death would occur at the beginning of year 1, affecting the following 5 years of the time horizon. Nevertheless, assumptions were validated by the expert committee and included in the sensitivity analysis to test the strength of the results. Fifth, the time horizon of the present analysis was set at 5 years from the diagnosis, yet a different time horizon could yield a different social and economic impact for each type of HbA_1c_ control. Nevertheless, following a conservative approach, a 5-year time horizon accounts for the minimum social value that would be generated with respect to longer time horizons. Finally, this study was tailored to the Spanish NHS; hence, results cannot be extrapolated to other countries. Notwithstanding, the results of the present study highlight the need for more comprehensive studies on the socioeconomic impact of a tight and early control of type 2 diabetes and may set the basis for future studies.

## Conclusion

In conclusion, the results suggest that maintaining tight and early control of type 2 diabetes can significantly reduce the impact of the disease on patients, the healthcare system, and society. Moreover, the need to improve clinical outcomes through tight and early control of type 2 diabetes has been confirmed in the present study, as evidenced by the clinical benefits of this intervention. Therefore, standard control of type 2 diabetes should not be the therapeutic aim, unless otherwise stated.

There is currently a large and growing prevalence of obesity (a major risk factor) and prediabetes within the population (33, 42–44). Moreover, underdiagnosis of these conditions has been reported (45), and the prevalence of type 2 diabetes is also on the rise due to the increase in life expectancy (46). These factors, combined with aging population, present a complicated challenge for meeting future healthcare requirements. In this regard, the healthcare budget should be used as an investment for which results are expected to occur in the medium to long term, as patients with controlled type 2 diabetes incur lower costs for the healthcare system compared to uncontrolled patients (47). Moreover, public administrations should promote the use of real-world data and economic evaluations to conduct studies that can guide clinical and health management decisions more accurately. A multidimensional intervention that unites patients and professionals, therapeutic tools, the healthcare system, administration, and society may be the most effective solution to the challenges we currently face.

The results of the present study reveal that optimizing the quality of care and efficiency in diabetes management is possible. These findings may be used to identify areas where tight and early control of type 2 diabetes could have a large impact through the creation of social value, which may guide decision-making and help focus the investment on improving disease management, health promotion, and quality of life. The sustainability of healthcare systems should be global and based on social, environmental, and economic sustainability (12).

## Data Availability

The raw data supporting the conclusions of this article will be made available by the authors, without undue reservation.

## Supplementary material

SUPPLEMENTARY FIGURE 1 Outcomes associated with each type of HbA1c within each area of analysis. **(A)** Complications. **(B)** Hospitalizations. **(C)** Quality of life. **(D)** Mortality.

SUPPLEMENTARY FIGURE 2 Economic impact by area of analysis and HbA1c control, per patient.
