# Supplementary figures and images for: Tight and early HbA1c control in patients with type 2 diabetes mellitus in Spain: quantifying the social value
Source: Front Public Health. 2025 Jul 11;13:1511108. doi: 10.3389/fpubh.2025.1511108 (PMC12291102; doi:10.3389/fpubh.2025.1511108)

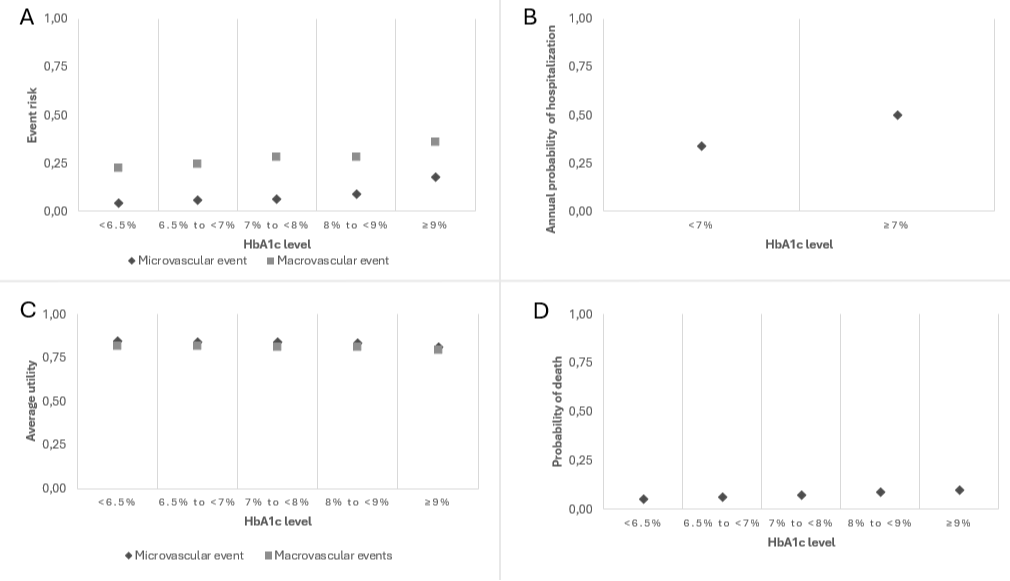

Supplement: SUPPLEMENTARY FIGURE 1 — Outcomes associated with each type of HbA1c within each area of analysis. (A) Complications. (B) Hospitalizations. (C) Quality of life. (D) Mortality. [file Image_1.png]

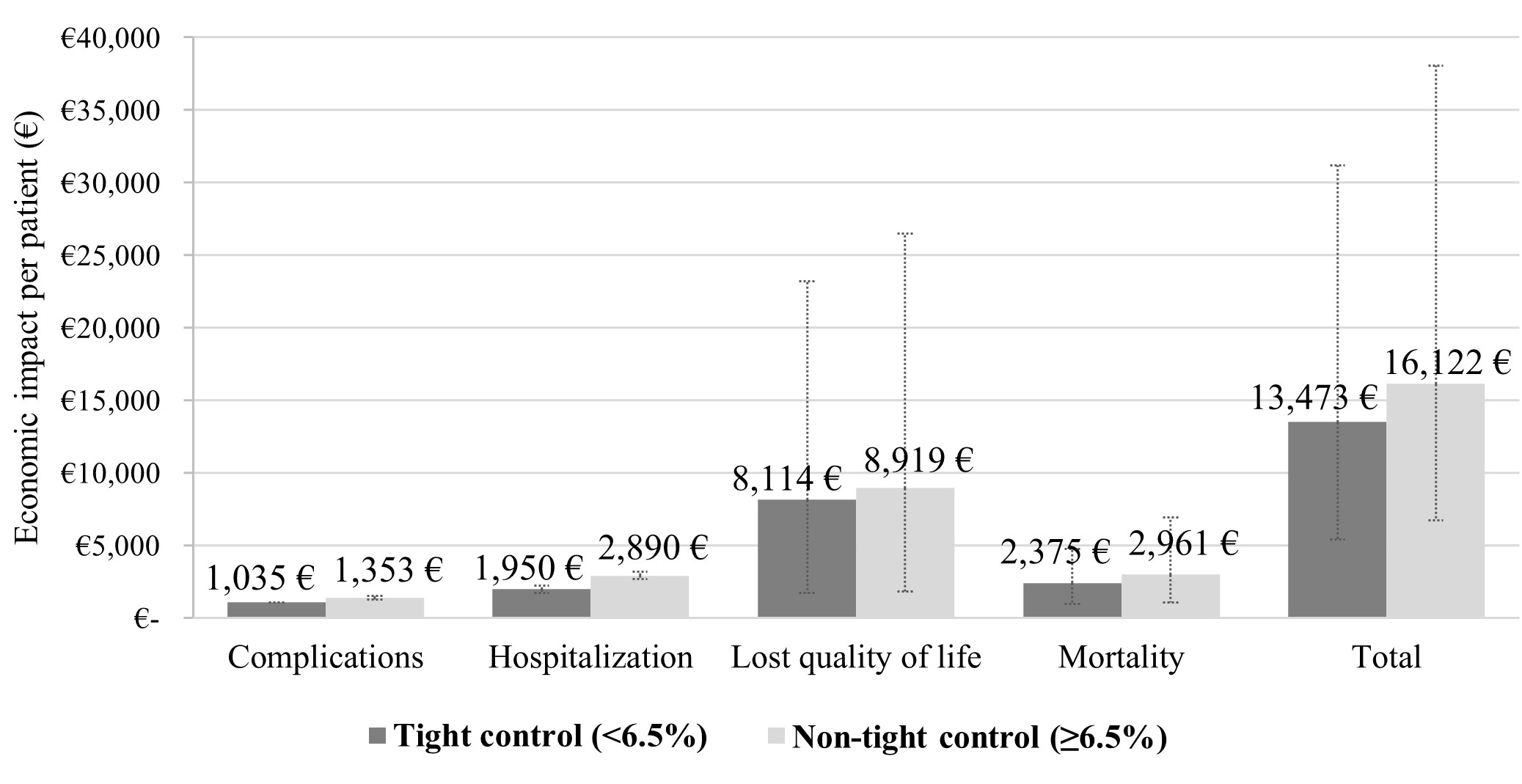

Supplement: SUPPLEMENTARY FIGURE 2 — Economic impact by area of analysis and HbA1c control, per patient. [file Image_2.jpeg]
