# Supplementary material for: Tight and early HbA1c control in patients with type 2 diabetes mellitus in Spain: quantifying the social value
Source: Front Public Health. 2025 Jul 11;13:1511108. doi: 10.3389/fpubh.2025.1511108 (PMC12291102; doi:10.3389/fpubh.2025.1511108)
Supplement: Supplementary file 3 [file Table_1.docx]

Supplementary Material

# Table 1. Data points included in the sensitivity analysis.

| **Areas of analysis and outcomes** | | **HbA_1c_ level** | **Lower limit** | **Base scenario** | **Upper limit** |
| --- | --- | --- | --- | --- | --- |
| **COMPLICATIONS** |  |  |  |  |  |
| *Probability of having a microvascular event in the first five years since diagnosis* (1) | | *<6.5%^a^* | *0.043* | *0.043* | *0.043* |
|  |  | *6.5% to <7%^b^* | *0.047* | *0.055* | *0.063* |
|  |  | *7% to <8%^b^* | *0.054* | *0.062* | *0.072* |
|  |  | *8% to <9%^b^* | *0.077* | *0.091* | *0.109* |
|  |  | *≥9%^b^* | *0.148* | *0.177* | *0.212* |
| *Average cost per hospitalization due to a microvascular event* (2) | | *All HbA_1c_ levels^c^* | *€ 4,294* | *€ 4,294* | *€ 4,294* |
| *Probability of having a macrovascular event in the first five years since diagnosis* (1) | | *<6.5%^a^* | *0.226* | *0.226* | *0.226* |
|  |  | *6.5% to <7%^b^* | *0.232* | *0.249* | *0.268* |
|  |  | *7% to <8%^b^* | *0.266* | *0.285* | *0.306* |
|  |  | *8% to <9%^b^* | *0.260* | *0.287* | *0.317* |
|  |  | *≥9%^b^* | *0.321* | *0.361* | *0.406* |
| *Average cost per hospitalization due to a macrovascular event* (2) | | *All HbA_1c_ levels^c^* | *€ 3,748* | *€ 3,748* | *€ 3,748* |
| **HOSPITALIZATIONS** |  |  |  |  |  |
| *Annual hospitalization probability not linked to micro or macrovascular events* (3) | | *<7%* | *0.297* | *0.340* | *0.384* |
|  |  | *≥7%* | *0.462* | *0.504* | *0.547* |
| *Average cost per hospitalization due to type 2 diabetes* (2) | | *All HbA_1c_ levels^c^* | *€ 2,406* | *€ 2,406* | *€ 2,406* |
| **LOSS OF QUALITY OF LIFE** | |  |  |  |  |
| *Average utility linked to microvascular events* (1,4) | | *<6.5%^d^* | *0.846* | *0.846* | *0.846* |
|  |  | *6.5% to <7%* | *0.845* | *0.843* | *0.841* |
|  |  | *7% to <8%* | *0.843* | *0.841* | *0.839* |
|  |  | *8% to <9%* | *0.838* | *0.834* | *0.830* |
|  |  | *≥9%* | *0.821* | *0.814* | *0.805* |
| *Average utility linked to macrovascular events* (1,4) | | *<6.5%^d^* | *0.820* | *0.820* | *0.820* |
|  |  | *6.5% to <7%* | *0.819* | *0.816* | *0.813* |
|  |  | *7% to <8%* | *0.813* | *0.810* | *0.807* |
|  |  | *8% to <9%* | *0.814* | *0.810* | *0.805* |
|  |  | *≥9%* | *0.804* | *0.798* | *0.791* |
| *Number of years affected by micro or macrovascular event since onset^e^* | | *All HbA_1c_ levels* | *1* | *2.5* | *5* |
| *Incremental cost-effectiveness ratio per QALY gained* (5) | | *All HbA_1c_ levels* | *€ 11,000* | *€ 21,000* | *€ 30,000* |
| **MORTALITY** | |  |  |  |  |
| *Probability of death in the first five years since diagnosis* (1) | | *<6.5%^a^* | *0.058* | *0.058* | *0.058* |
|  |  | *6.5% to <7%^b^* | *0.055* | *0.062* | *0.071* |
|  |  | *7% to <8%^b^* | *0.063* | *0.072* | *0.082* |
|  |  | *8% to <9%^b^* | *0.075* | *0.091* | *0.109* |
|  |  | *≥9%^b^* | *0.081* | *0.102* | *0.129* |
| *Year in which death occurs since onset^f^* | | *All HbA_1c_ levels* | *5* | *2.5* | *1* |
| *Average annual earnings per worker in Spain* (6) | | *All HbA_1c_ levels^c^* | *€ 22,838* | *€ 22,838* | *€ 22,838* |

*^a^*Data points are reference values used to estimate probabilities for glycemic levels ≥6.5% by applying the corresponding adjusted hazard ratios to the reference value for glycemic level <6.5%, hence, 95% confidence intervals were only available for HbA_1c_ levels ≥6.5%. *^b^*Assumption based on the corresponding adjusted hazard ratio with respect to the reference value for glycemic level <6.5%. *^c^*Data points were national estimates that were not varied between sensitivity analysis scenarios as 95% confidence intervals were not available. *^d^*Data points related to reference microvascular and macrovascular event probabilities that were not varied between sensitivity analysis scenarios (refer to note *^a^*). *^e^*Assumption. The lower limit is 1, which means that the event occurs in year 5 of the time horizon, with a loss of quality of life for 1 year; the upper limit is 5, meaning that the event occurs in year 1 of the time horizon, with a loss of quality of life for 5 years. *^f^*Assumption. The lower limit is 5, which implies a loss of work productivity for 1 year; the upper limit is 1, meaning a loss of work productivity for 5 years. Abbreviations: HbA_1c_, hemoglobin A1c; QALY, quality-adjusted life years.

**Table 2.** Economic impact and social value by area of analysis and HbA_1c_ control, per patient and in Spain.

| **Area of analysis** | **Economic impact / social value** | **HbA_1c_ control** | **PER PATIENT (€)** | | | **SPAIN (€)** | | |
| --- | --- | --- | --- | --- | --- | --- | --- | --- |
|  |  |  | **Lower limit** | **Base scenario** | **Upper limit** | **Lower limit** | **Base scenario** | **Upper limit** |
| **COMPLICATIONS** | Economic impact | Tight control*^a^* | *1,034.5* | *1,034.5* | *1,034.5* | *77,568,395* | *77,568,395* | *77,568,395* |
|  |  | Non-tight control | *1,227.7* | *1,352.5* | *1,492.1* | *85,801,523* | *94,526,210* | *104,278,427* |
|  | **Social value** |  | ***193.2*** | ***318.1*** | ***457.6*** | ***13,505,346*** | ***22,230,033*** | ***31,982,250*** |
| **HOSPITALIZATION** | Economic impact | Tight control | *1,703.0* | *1,949.6* | *2,201.9* | *127,700,487* | *146,189,110* | *165,107,700* |
|  |  | Non-tight control | *2,649.1* | *2,890.0* | *3,136.5* | *185,143,558* | *201,974,790* | *219,206,766* |
|  | **Social value** |  | ***946.1*** | ***940.4*** | ***934.7*** | ***66,122,699*** | ***65,721,955*** | ***65,321,212*** |
| **LOSS OF QUALITY OF LIFE** | Economic impact | Tight control | *1,700.1* | *8,114.2* | *23,183.3* | *127,482,459* | *608,439,011* | *1,738,397,173* |
|  |  | Non-tight control | *1,803.2* | *8,918.7* | *26,484.7* | *126,022,034* | *623,310,065* | *1,850,967,034* |
|  | **Social value** |  | ***103.1*** | ***804.5*** | ***3,301.4*** | ***7,204,384*** | ***56,225,827*** | ***230,726,354*** |
| **MORTALITY** | Economic impact | Tight control | *950.0* | *2,374.9* | *4,749.8* | *71,233,113* | *178,082,783* | *356,165,566* |
|  |  | Non-tight control | *1,016.5* | *2,961.1* | *6,907.9* | *71,038,706* | *206,944,660* | *482,777,938* |
|  | **Social value** |  | ***66.5*** | ***586.2*** | ***2,158.0*** | ***4,647,210*** | ***40,965,921*** | ***150,820,461*** |
| **TOTAL** | Economic impact | Tight control | *5,387.5* | *13,473.1* | *31,169.5* | *403,984,455* | *1,010,279,299* | *2,337,238,835* |
|  |  | Non-tight control | *6,696.5* | *16,122.3* | *38,021.1* | *468,005,820* | *1,126,755,724* | *2,657,230,165* |
|  | **Social value** |  | ***1,308.9*** | ***2,649.1*** | ***6,851.7*** | ***91,479,639*** | ***185,143,736*** | ***478,850,276*** |

Note: the social value at a population level corresponds to the total reduction of the economic impact that could be obtained if all patients with a non-tight control in Spain had tight control. *^a^*The data does not vary between sensitivity analysis scenarios. Abbreviations: HbA_1c_, hemoglobin A1c.

**References**

1. Laiteerapong N, Ham SA, Gao Y, Moffet HH, Liu JY, Huang ES, et al. The Legacy Effect in Type 2 Diabetes: Impact of Early Glycemic Control on Future Complications (The Diabetes & Aging Study). Diabetes Care [Internet]. 2019 Mar [cited 2022 Apr 26];42(3):416–26. Available from: https://www.ncbi.nlm.nih.gov/pmc/articles/PMC6385699/

2. Ministerio de Sanidad. Ministerio de Sanidad. Subdirección General de Información Sanitaria. Registro de Actividad de Atención Especializada – RAE-CMBD. 2020.

3. Schneider ALC, Kalyani RR, Golden S, Stearns SC, Wruck L, Yeh HC, et al. Diabetes and Prediabetes and Risk of Hospitalization: The Atherosclerosis Risk in Communities (ARIC) Study. Diabetes Care [Internet]. 2016 Mar 7 [cited 2022 May 3];39(5):772–9. Available from: https://doi.org/10.2337/dc15-1335

4. Instituto Nacional de Estadística. Encuesta Nacional de Salud 2011-2012. Cuestionario de Adultos [Internet]. 2012 [cited 2018 Oct 30]. Available from: https://www.mscbs.gob.es/estadisticas/microdatos.do

5. Ortega Eslava A. Guía de evaluación económica e impacto presupuestario en los informes de evaluación de medicamentos. Madrid: SEFH, Sociedad Española de Farmacia Hospitalaria; 2017.

6. Instituto Nacional de Estadística. INE. 2020 [cited 2022 Jun 28]. Encuesta anual de estructura salarial. Año 2020. Available from: https://www.ine.es/dyngs/INEbase/es/operacion.htm?c=Estadistica_C&cid=1254736177025&menu=ultiDatos&idp=1254735976596
